# Historic Floodplain Meadows in the Landscape: Investigating Anthropogenic Habitats Important for Nature Conservation, Carbon Sequestration and Flood Attenuation

**DOI:** 10.1007/s00267-026-02396-2

**Published:** 2026-02-18

**Authors:** Antony Firth, Emma Firth, Emma Rothero, David Gowing

**Affiliations:** 1 Fjordr Ltd., Windover House, St. Ann Street, Salisbury, UK; 2 School of Environment, Earth and Ecosystem Science, The Open University, Milton Keynes, UK

**Keywords:** Floodplain, Meadow, Agriculture, Landscape, Nature recovery, Heritage

## Abstract

In contemporary river valley floors, floodplain meadows are associated with rare grassland plant communities that are important for their conservation value. This article outlines a desk-based method to identify, map and record floodplain meadows as historic features in the landscape, based on physical forms embodying agricultural practices dating back to the medieval period. Mapping the former location, extent and distribution of floodplain meadows independently of the survival of habitat provides transparent, place-based evidence of the decline of floodplain meadow habitats. It also supports restoration of floodplain meadows based on former locations that date back many centuries. The article summarises catchment-based studies carried out across England and Wales between 2017 and 2024. The methodology is GIS-based and uses a variety of base layers, historic landscape sources such as maps and lidar, and non-mappable documentary and published sources, all of which are readily accessible to users. In total, 373 historic floodplain meadows in seven catchments were identified, recorded, and added to a freely available online map. The results show that floodplain meadows were present in the historic landscape in many parts of England and Wales and suggest that even where meadows were situated on the riverine peripheries of manors or parishes, they were central to the structure of agricultural societies and the landscapes to which they gave rise. The approach that has been developed is relevant throughout the geographic range of floodplain meadows, reflecting at least the extent of medieval open field agriculture in Europe. However, the methodology may also be relevant to the restoration of anthropogenic habitats across the globe that are of high importance for nature conservation, climate mitigation and adaptation, where traces interpretable from the historic landscape are more extensive than the survival of the habitat itself.

## Introduction

### Background

Floodplain meadows have distinctive, diverse communities of plants that sequester carbon, attenuate flooding, and provide important habitats. These important benefits arise from plant communities that are fundamentally anthropogenic in character: a consequence of agricultural practices directed to the production of hay and grazing of high quantity and quality over centuries (Schaich et al. 2011; McGinlay et al. 2016).

However, these agricultural practices changed, resulting in massive loss of floodplain meadows alongside other types of species-rich grassland both in the UK and on the European continent. Agricultural improvement from the 1930s including drainage, ploughing, use of artificial fertiliser, intensive grazing and herbicide application reduced the overall extent of grassland, and—where grassland persists—reduced its conservation value. By 1984, the total extent of unimproved lowland grassland in England and Wales was just 0.6 M ha, an overall decline of 92% (Fuller 1987, 289). Unimproved lowland pasture, excluding rough grasslands, had declined to 0.2 M ha and ‘It may only be a small proportion of the 0.2 M ha of “unimproved” grasslands which still retain an especially interesting or diverse flora and/or fauna’ (Fuller 1987, 297). Similarly, Krause et al. report that in north Germany, species-rich mesic meadows decreased by 83.6% between the 1950/1960s and 2008 in favour of intensively managed grasslands and arable fields (Krause et al. 2011, 2352). Fragmentation and lack of connectivity is an important negative factor in addition to reduction in extent (Krause et al. 2011, 2359).

The diverse grassland communities created by the management of floodplain meadows are now rare and of high nature conservation importance across Europe. Moist or wet, mesotrophic to eutrophic hay meadows (Eunis habitat type E2.14 and E3.4a, EEA 2019^1^) are classed as Endangered in the European Red List of habitats (European Commission 2016). There is strong interest (Vinther and Hald 2000; Hölzel and Otte 2003) in conserving surviving floodplain meadows and, in re-creating floodplain meadows for their habitat value and other ecosystem services (Lawson et al. 2018; Sommer et al. 2023).

The case for restoration is supported by better understanding the original extent of floodplain meadows overall, but also knowing the specific localities where they were present. Recognising their earlier presence provides reassurance that the environment is suitable for restoration. Typically, the location of floodplain meadows has been established by identifying the distinctive plant communities where they still survive. However, these surviving remnants represent a tiny fraction of their original extent and offer a potentially misleading indication of their earlier distribution: the lack of evidence for the former location and extent of floodplain meadows is a barrier to their restoration.

This article presents an approach that focusses not on surviving habitats but on physical and documented traces in the landscape of the agricultural practices that gave rise to them. In contrast to instances of surviving habitat, the physical and documented traces of floodplain meadows appear to be much more extensive and broadly distributed. A further important advantage is that physical and documentary traces are spatially and, to a degree, chronologically specific whereas, as Krause et al. note, ‘historically spatially explicit vegetation data are rare’ (Krause et al. 2011, 2349). Traces in the landscape also extend much further back in time than extensive vegetation recording: the starting point for Fuller (1987) is a survey in 1930–38 whilst the landscape evidence explored here stretches back centuries, even millennia.

The approach outlined here informs our understanding of the original extent and potential for restoration, but paying attention to these physical and documented traces in the landscape offers further benefits: it has the potential to identify features such as boundaries, drains and droves that still survive from floodplain meadows as heritage assets^2^ despite their great age; it underlines the relationship between floodplain meadows and the communities whose traditional practices created them; and provides a tangible focus in the landscape for engaging with farmers, land owners, catchment managers and the public about floodplain meadows and their restoration. The approach is complementary to research into the Traditional Ecological Knowledge (TEK) of hay meadows via traditional practices, oral histories, historical narratives, and cultural representations (Burton and Riley 2018; Bele et al. 2024; Moore-Colyer 2021), seeking knowledge of meadow management that is embodied in the landscape itself. This approach is relevant throughout the geographic range of floodplain meadows, reflecting at least the extent of medieval open field agriculture in Europe. However, it is also relevant to the restoration of anthropogenic habitats of high conservation importance across the globe where traces interpretable from the historic landscape are more extensive than the current survival of the habitat itself.

### Origins of this Approach

In recent years, Fjordr Ltd has developed a GIS-based methodology for flagging the historic character of rivers and other watercourses. The methodology was developed initially through two projects funded by Historic England: one on the Dorset Stour (Firth and Firth 2020) and one on the River Culm in Devon (Firth and Firth 2024b). Evidence of human activity or interventions related to the watercourse are mapped as Historic Watercourse Polygons (HWPs) and core information such as period, description and sources is recorded. The rationale and implementation of the method is set out in the project report for the Dorset Stour.

Mapping and recording HWPs enables synthesis of diverse sources – including historic maps, lidar (light detection and ranging) data and existing archaeological and historical records – into a single layer. The methodology is strategic in intent and is applied at catchment scales to produce a GIS layer that can be used by catchment managers to alert them to the presence of historic features. The HWPs identified encompass numerous different types and themes: watermills; crossings; waterfronts; palaeochannels and so on.

In the historic mapping for both the Dorset Stour and the River Culm, distinctive forms of field alongside the rivers were identified, which were termed ‘funnel-shaped meadows’ in the GIS because of their funnel-shaped entrances that connect open areas of floodplain to green lanes and droves. They echo the funnel-shaped entrances seen where roads and droves enter a common to facilitate the movement of livestock (Rippon and Clark, 2004, p. 21; Ryder, 2013, pp. 110–112). These funnel-shaped meadows are distinct from the fields surrounding them, which in turn seem to respect their often sinuous, irregular boundaries. This suggested that the funnel-shaped meadows pre-date the rectilinear enclosed fields that abut them. Furthermore, on tithe maps, the funnel-shaped meadows often have ‘mead’ names and are sub-divided into multiple strips – demarcated by dotted lines – which fit within the boundaries of the meadow. It became obvious that the ‘funnel-shaped meadows’ apparent on the Dorset Stour and River Culm were a form of floodplain meadow managed in common for shared benefits.

### Medieval History of Floodplain Meadows

The distinctive form of floodplain meadows arises from how they were managed: as commons used to produce one or sometimes two cuts of hay followed by grazing of livestock on the aftermath. Rights to hay were allocated to commoners by way of strips, referred to here as doles; and the movement of livestock to and from common land gave rise to their funnel-shaped connection to green lanes and droveways.

The longevity of these common meadows—apparently surviving in use from the medieval period until at least the 1840s, when tithe maps still show the dole system—reflects the length of time invested in attaining a sustainable productive meadow, and its continuing value. Rackham (1997, 332) observes: ‘Making good grassland, although easy, took some years to achieve, and hence farmers and their landlords traditionally regarded pasture, and especially meadow, as a fixture: they were reluctant to plough it up even if this appeared to be profitable’. The huge importance of a good hay crop for the functioning and prosperity of agricultural communities caused the form of these meadows to survive, resulting—with other elements of medieval boundaries and field systems—in their incorporation into the post medieval and modern landscape whilst new field systems and patterns of land use evolved around them. Consequently, the funnel-shaped floodplain meadows that are evident in nineteenth century and modern mapping—and which are still evident on the ground today in some places—are the remnants of floodplain meadows that are medieval in origin.

The importance and value of meadows to the medieval and later rural economy is set out by Hall (2014, pp. 17–20), who notes that meadows were present both in the floodplains of major rivers but also adjacent to smaller tributaries. As with the contemporary arable land, which comprised strips within open fields that gave rise to distinctive ridge and furrow earthworks, meadows were also demarcated in strips referred to using various terms such as ‘lots’ or ‘doles’, which could be marked out on the ground with poles or stones (Rackham, 1997, pp. 337–8; Hall, 2014, pp. 18–19; McDonald, 2007, pp. 73–82; Bowden et al., 2009, pp. 27–28). The strips of meadow were allocated each year using various means, such as ‘drawing lots’. Such practices lasted—in some cases—into the 20th century: meadow was being assigned in Yarnton and Begbroke, Oxfordshire, in 1910 using balls marked with the names of residents from the 13^th^ century; and the area of meadow allocated approximated to that referred to in the Domesday survey of 1086 (Hall, 2014, p. 19). In many places, the management of floodplain meadows as commons and their systems of allocation were variously replaced in the post-medieval and modern periods by private ownership of strips, of entire former commons, and of enclosed fields taking the place of former commons (Pearson and Soar, 2018).

It is likely that systems of common meadows originated in the early medieval period, predating the Domesday survey of AD 1086 by several hundred years: King Ine’s law code for Wessex included clauses on damage to common crops or grass including ‘common meadow or other land divided into shares’ in the late seventh or early eighth century (Rippon 2014, p. 19; Williamson, 2015, p. 177). Use of floodplains for meadows appears to have intensified from the eighth century and seems to be associated in particular with the development from dispersed to nucleated settlements, with the intense collaboration required by haymaking perhaps even being one of the stimuli for people to cluster, especially where the topography and hydrology was favourable for meadows (Williamson, 2015, pp. 201–204). Williamson makes the further point that the link between the availability of land for meadows and the location of nucleated settlements is not diminished by meadows being at a distance from settlements, as a short walk would be outweighed by being able to organise labour and equipment from a large village (Williamson, 2015, p. 204). This helpfully underlines the point that the relationship between floodplain meadows and their communities is fundamental, embodied in the routes – droveways – that both connected them and contributed to their distinctive funnel-shaped forms.

There seem to be strong resonances between the management of floodplain meadows and the management of the Anglo-Saxon Fenland set out by Oosthuizen (2017). She makes the case that fenlands were not marginal or unproductive, but a source of prosperity based on understanding and manipulating water and careful management of meadows for hay and grazing through rights of common. Movement of people and animals to make use of the fenland landscape as a whole were also critical. It may not be unreasonable to regard floodplain meadows as Oosthuizen sees the fens: perhaps on a smaller scale but similarly central to the prosperity of settlements rather than at the margin, as their location at the river bound edges of parishes might be interpreted.

### Antecedents: the Prehistory of Meadows in Floodplains

Archaeological evidence indicates that the use of floodplains as meadows for haymaking may stretch even further back in time. Investigations in the Upper Thames suggest that the floodplain was used for pasture in the Iron Age, with hay meadows appearing in the early Roman period, though they may not have been widespread (Booth et al., 2007, p. 22). McDonald pushes the maintenance of rough grazing at Port Meadow, Oxford, back to the Bronze Age, but suggests there is as yet no direct evidence for hay meadow as early as the Iron Age (McDonald, 2007, pp. 64–65). Booth et al. note that the population declined in the post Roman period and the intensity of agricultural activity with it, but the floodplain was not abandoned: use for grazing by domestic animals precluded woodland regeneration (Booth et al., 2007, p. 30). In the mid to late Saxon period (AD 650-1000) grassland in the floodplain was increasingly managed as hay meadow (Booth et al., 2007, pp. 30–31).

Investigations at Thornhill Farm near the Thames upstream of Lechlade echo this interpretation, with evidence for a hay meadow potentially contemporary with the site in the early Roman period (Jennings et al., 2004, p. 144). The site overall is interpreted as a specialised stock raising centre in the late Iron Age and early Roman periods, including a funnel-shaped track or droveway dating to AD 50-100 (Jennings et al., 2004, pp. 150; Fig. 3.10). The Thornhill Farm site might have been part of a wider intensification that included the development of specialist pastoral farms in the floodplain in the late Iron Age, reflecting pressures and developments in Britain that continued into the Roman period. Although the site at Thornhill Farm was apparently unaffected by the Roman conquest, the landscape was radically reorganised in the early 2^nd^ century AD associated in particular with the cultivation of hay meadows, perhaps as part of a more centrally organised estate (Jennings et al., 2004, p. 158).

The earliest evidence of hay meadows in Britain is indicated by analysis of waterlogged plants from features associated with the late Iron Age oppidum (a large fortified settlement) at Silchester in the catchment of the River Kennet, itself a tributary of the Thames (Lodwick 2017). To Lodwick, evidence of hay in former wells in an urban context suggests the organisation of the community beyond individual households, mobilising rapidly to harvest and make hay and to transport it to the oppidum: the agricultural innovation of managing meadows for hay may have been a consequence of communities aggregating in larger settlements prior to the Roman conquest (Lodwick 2017, 216).

The capacity of floodplains for specialised stock raising, including the production of hay, in the late Iron Age and Roman periods may have been important in the other catchments our investigations have explored. The Dorset Stour is characterised by a string of Iron Age hillforts and other major sites close to the river: floodplain meadows have been identified immediately adjacent to both Dudsbury Camp and Hod Hill. Similarly, Dolbury Hillfort overlooks the floodplain of the Culm at Killerton, where the vestiges of floodplain meadows are still apparent in field boundaries. It is perhaps too great a stretch to suggest that the funnel-shaped floodplain meadows still visible in their catchments are survivals from the Iron Age, but it seems reasonable to suggest continuities in the use of the floodplain for meadow – in different forms and places – stretching back over two millennia; and this immense timescale of human management of floodplain habitats must surely be relevant to our management of floodplain habitats in future.

### Other Types of Meadows in Floodplains

Floodplain meadows can be distinguished from other types of meadows in and near floodplains in which the management of water also plays a major role. The general term ‘water meadow’ encompasses the complex systems of interlocking feeder and drainage channels constructed predominantly in the seventeenth and eighteenth centuries better referred to as bedwork water meadows. Bedworks share characteristics with catchworks, which are hillside systems that rely on feeder channels constructed along contours. Both bedworks and catchworks are referred to as ‘floating downward’, bringing water from a river, tributary, or spring upstream; distributing the water as a shallow, constantly moving sheet; and collecting it in drains to return to the watercourse (Cook and Williamson 2007; Historic England 2018).

Bedworks and catchworks required extensive re-modelling and construction of channels. Constructing and maintaining these systems was intensive, but despite the effort and cost, the anticipated increase in productivity caused huge uptake among agricultural improvers and they were typically used through to the late nineteenth and early twentieth century.

Some meadows in the floodplain were irrigated artificially before the introduction of bedworks and catchworks. Water was distributed onto meadows by raising water levels in watercourses with weirs and providing channels to direct the water across the floodplain. This approach is known as ‘floating upward’ and is documented prior to the seventeenth century, potentially originating in the medieval period: Historic England notes that it is ‘barely represented in the archaeological record, since their remains are extremely difficult to identify’ (Historic England, 2018, pp. 2, 15). However, on the River Culm an early seventeenth century map in Devon Archives^3^ clearly illustrates a ‘floating upward’ system around Fulford Water—between Cullompton and Halberton—comprising multiple weirs and channels. Although earlier than bedworks and catchworks, these floating upwards systems are still based around artificially raising water levels and distributing water onto meadows by modifying or constructing channels. Consequently, floating upward systems contrast with floodplain meadows as considered here, which function through seasonal flooding rather than using feeder channels.

Although flooding naturally, floodplain meadows sometimes have a back drain along their landward boundary to assist with drainage, catching groundwater seepage from the hillslope to avoid the meadow becoming too wet through the growing season (they are often referred to as ‘catch-drains’, especially in Yorkshire). In earlier periods when the floodplain was not bordered by enclosed land, these drains may also have served as a ‘watery fence’ (Williamson 2002, 121) to prevent animals straying.

Our focus on the physical form and features of floodplain meadows underpins their distinction from a further type of meadow in floodplains, which is the use of conventional fields to produce hay. Floodplain meadows managed in common have distinct physical features integral to their function and character, but this does not preclude meadows being maintained on glebe or other privately held land in the floodplain. However, where the form of fields in the floodplain used as meadows is undistinguished, the longevity of the habitat is indeterminable: it can only be identified as meadow if its cultivation has been recorded in historic documents such as tithe apportionments and is valid only for that point in time. Our focus here is on floodplain meadows that were managed in common for so long that they shaped the landscape even after the habitat was extinguished.

## Mapping and Recording Floodplain Meadows

Having mapped several funnel-shaped floodplain meadows alongside the Dorset Stour and River Culm and noted their possible antiquity, Fjordr Ltd. and the Floodplain Meadows Partnership (FMP) explored the development of a methodology that could be used more widely in clarifying the historic extent of floodplain meadows to support nature-conservation objectives. Alongside this, the relationship between floodplain meadows and parishes, and the potential relationship between floodplain meadows and medieval populations was also investigated. The potential for this historic dimension to engage stakeholders and the public was also recognised, including in stimulating local investigations by community groups involved in floodplain-meadow conservation: the methods and results of investigations of historic floodplain meadows have featured in numerous online and in-person presentations by Fjordr Ltd and FMP.

**Fig. 1:**
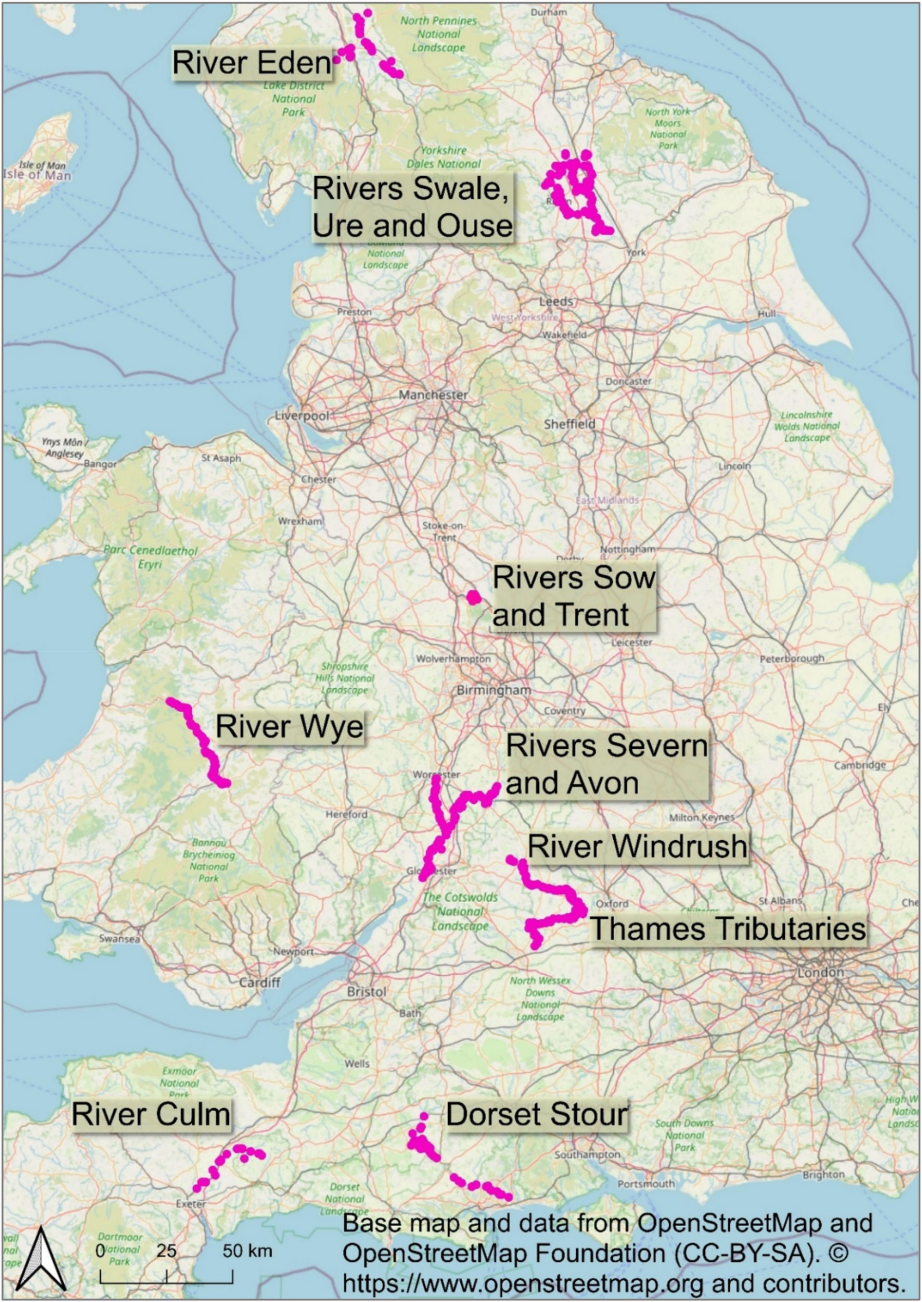
Map of England and Wales showing locations of floodplain meadows on river catchments and parts of river catchments referred to in the text. For further detail see https://floodplainmeadows.org.uk/discover/learn/history

To develop the method, the HWP assessment on the Dorset Stour was re-visited, alongside trialling the method along the tributaries of the Thames (Firth and Firth 2022). Further catchments have subsequently been investigated expressly for floodplain meadows, including: the Severn and Avon (Gloucestershire, England) (Firth and Firth 2024a); the Swale, Ure and Ouse (Yorkshire, England) (Firth and Firth 2023b); the Windrush (Thames, England) (Firth and Firth 2024b); and the upper reaches of the Wye (Powys, Wales) (Firth and Firth 2024c). Floodplain meadows have also been identified in the course of wider historic watercourse studies on the River Eden and its tributaries in Cumbria (England), and the Rivers Sow and Trent at Shugborough in Staffordshire (England) (Firth and Firth 2024a), as well as on the Dorset Stour and River Culm (Devon, England) (Firth and Firth 2021; Firth and Firth 2024b) mentioned previously (Fig. 1).

**Fig. 2:**
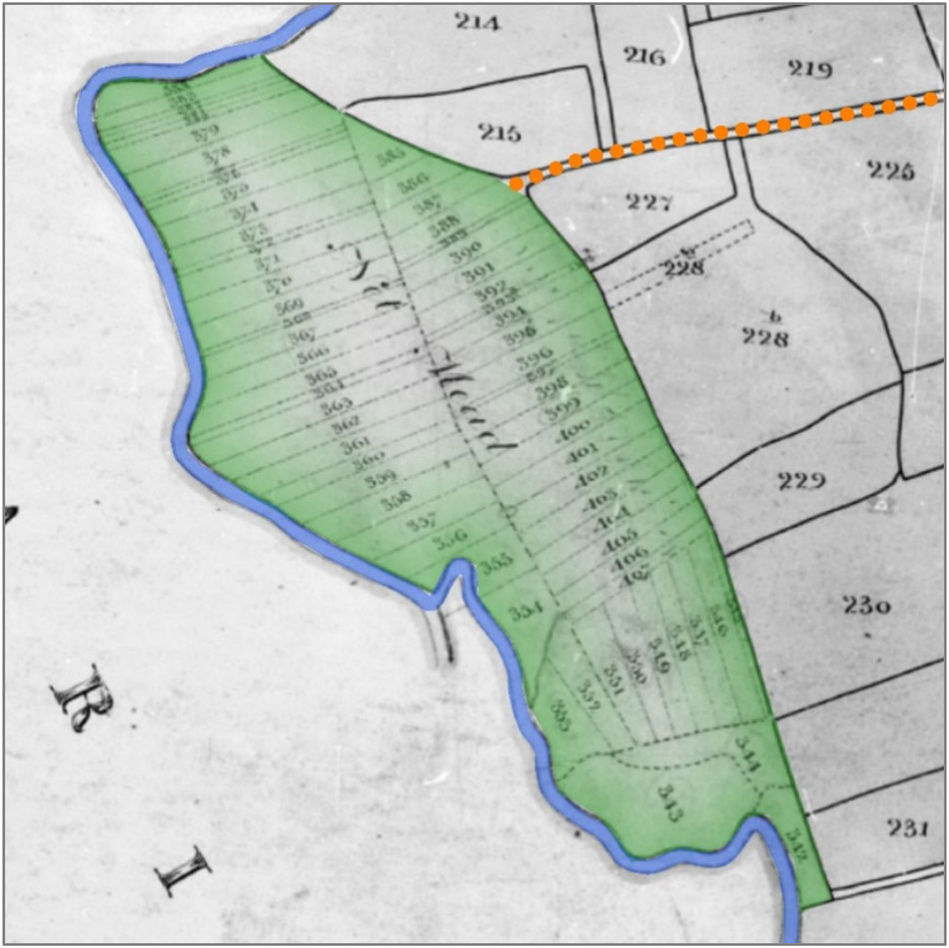
Net Mead, Dorset Stour, illustrating diagnostic features. Droveway (orange dots) connects to floodplain meadow (green tint) via a funnel-shaped entrance. The floodplain meadow is bounded on one side by the river (blue) and on its other sides by curving boundaries flaring out from the funnel-shape: these landward boundaries are respected by abutting (later) field boundaries and may follow the edge of the floodplain. The floodplain meadow is subdivided into doles marked by dotted lines on the original, and has a meadow name (‘mead’). Tithe map of Child Okeford (parish), Dorset, 1839. The National Archives, reference IR 30/10/60. © Crown Copyright Images reproduced by courtesy of The National Archives, London, England. www.NationalArchives.gov.uk & www.TheGenealogist.co.uk

**Fig. 3:**
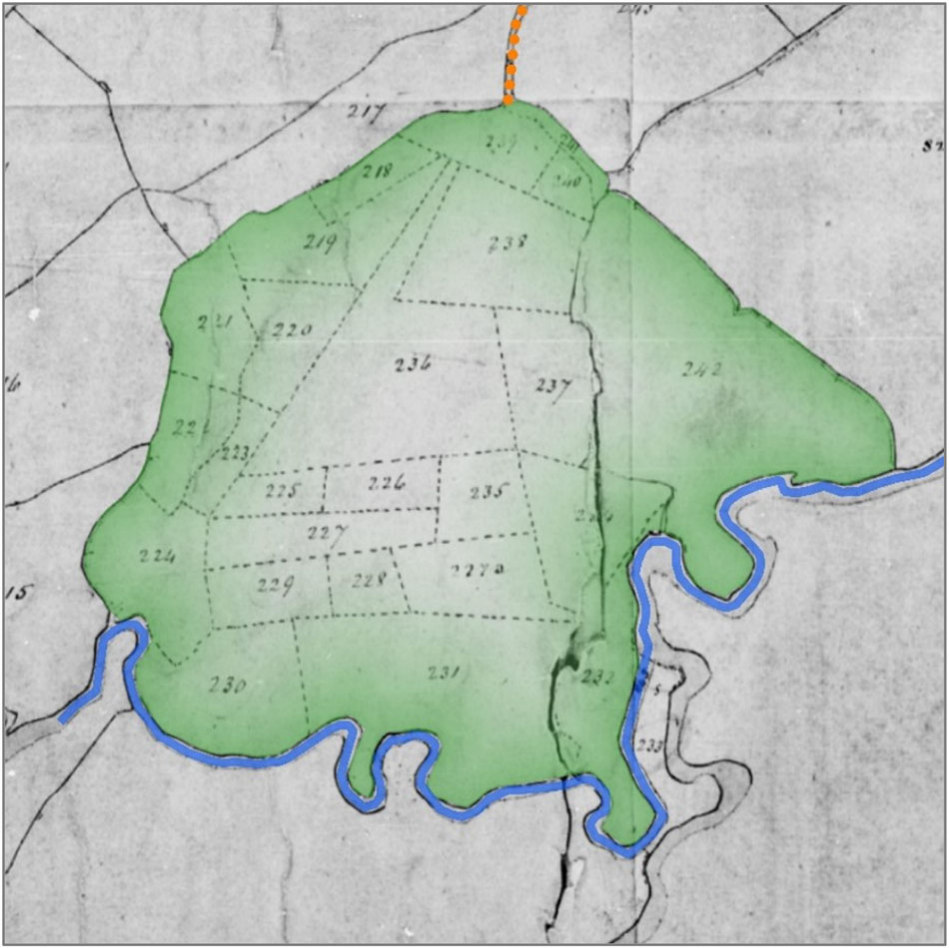
Floodplain meadow at Silverton, River Culm. Droveway (orange dots) enters the floodplain meadow (green tint) through a funnel-shape. The floodplain meadow is sub-dividend into doles, marked by dotted lines on the original. Tithe map of Silverton (parish), Devon, 1842. The National Archives, reference IR 30/9/373. © Crown Copyright Images reproduced by courtesy of The National Archives, London, England. www.NationalArchives.gov.uk & www.TheGenealogist.co.uk

**Fig. 4:**
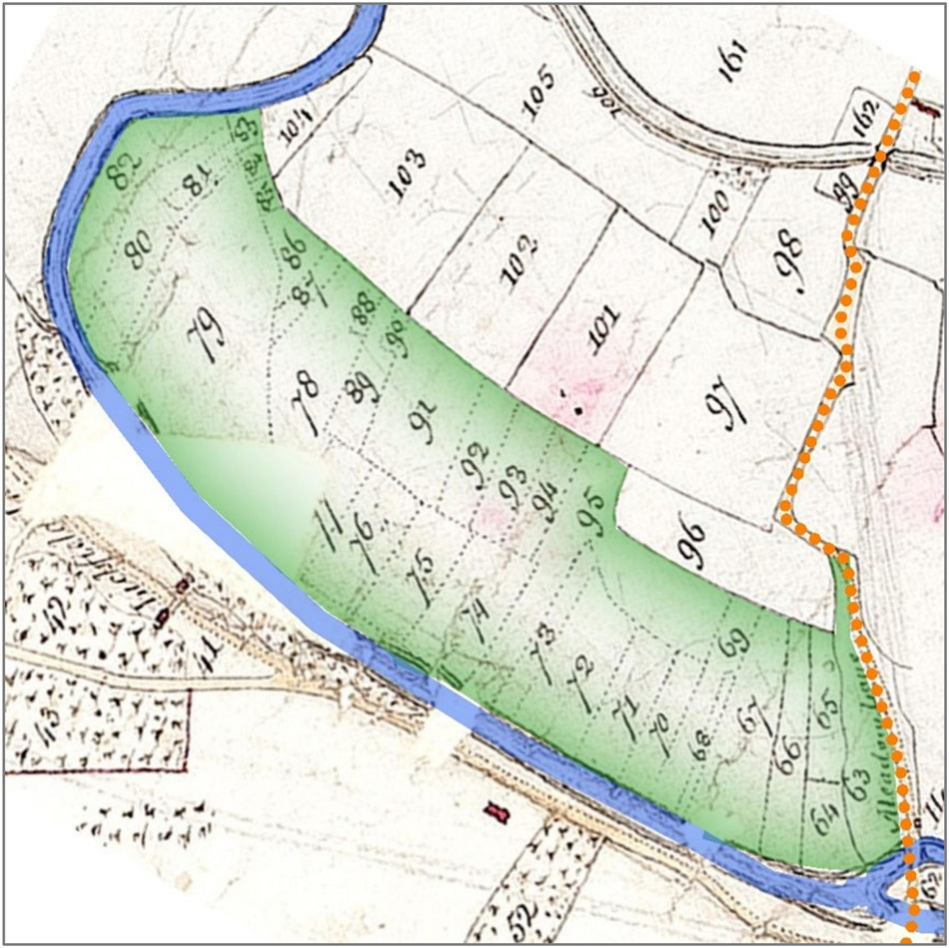
Floodplain meadow (green tint) at Little Haywood, River Trent. ‘Meadow Lane’ is marked with orange dots, providing a funnel-shaped entrance adjacent to plot 96: Meadow Lane continues through the floodplain meadow to Haywood Ford. Doles are shown with dotted lines on the original. Landward of the floodplain meadow, the rectilinear enclosed fields appear to have encroached on the common meadow as there is a relic of an earlier funnel-shaped entrance adjacent to plots 98 and 97. These rectilinear plots reflect the presence of a bedwork water meadow. Map of Shugborough and Great and Little Haywood, c. 1805. Staffordshire and Stoke on Trent Archive Service, reference D615/M/6/45. Reproduced courtesy of Staffordshire Record Office

Revisiting the Dorset Stour indicated the presence of certain diagnostic features that, in combination, indicate floodplain meadows formerly managed as commons (Fig. 2, Fig. 3, Fig. 4):

- The floodplain meadow is situated mostly in the modern floodplain.
- The floodplain meadow has one or more funnel-shaped entrances, usually coming off a droveway or a trackway leading from a settlement.
- One of its boundaries (usually the longest) is bounded by the river, while two sides flare out from the funnel-shaped entrance.
- On tithe maps, the floodplain meadow may be subdivided into strips (doles) that respect the irregular nature of the boundaries of the floodplain meadow but are themselves regular in shape and size.
- Where there are enclosed fields within the extent of the floodplain meadow, these fields are in strip form (reflecting individual or multiple doles).
- The ‘state of cultivation’ recorded in tithe apportionments is usually either meadow or pasture, but very rarely arable.
- In tithe apportionments and other documentary sources the fields have plot names associated with meadows, such as mead or ham.

Not all these features are clearly present in every case: the evidence might be limited to one or two features or be otherwise ambiguous. Accordingly, the methodology includes a ‘confidence level’ (1 = low confidence; 5 = high confidence) ascribed to each meadow that has been identified and mapped, informed by the diagnostic features listed above and based on professional judgement.

As well as the diagnostic features, there also appears to be an association between floodplain meadows and footpaths, reflecting their former status as commons and facilitated by the onward connections provided by droves. This association can be observed on some estate maps, on some historic Ordnance Survey (OS) maps and even as rights of way: but local histories of access in the countryside have their own complexities, so the presence of footpaths is at best contextual when seeking to identify floodplain meadows, rather than a firm indicator. Similarly, floodplain meadows may be associated with cultivated wet woodland such as osier beds^4^.

As case studies were added, GIS data were aggregated to provide an online ‘Map of Historic Sites’, available via the Floodplain Meadows Partnership website^5^. The online map shows the location and extent of historic floodplain meadows identified to date with their recorded attributes, which enable the map to be searched and filtered. Data continue to be added to the online map.

## Results

To February 2024 the aggregated data underpinning the online map^6^ totalled 373 floodplain meadows in study areas across seven catchments (Table 1).

**Table 1:** Count of floodplain meadows recorded in each study area, with level of confidence

Catchment	Total	Level of Confidence – Count				
		**1**	**2**	**3**	**4**	**5**
Dorset Stour	41		4	6	14	17
River Culm	19		1	4	4	10
River Eden	31	2	15	8	5	1
Severn and Avon	81		10	32	23	16
Swale, Ure and Ouse	106		33	44	23	6
Thames Tributaries	37		4	8	14	11
Windrush	58	1	17	35	5	
**Total**	**373**	**3**	**84**	**137**	**88**	**61**

Level of confidence is included here to underline the interpretative character of this approach: very few identified floodplain meadows have been ranked at the lowest level of confidence, but otherwise the totals exhibit a relatively normal bell curve. Differences between catchments in confidence levels reflect variability in the availability and quality of sources and in the degradation of floodplain meadows (e.g. by enclosure—see below) by the time they appear in sources.

The commonality of the diagnostic features for floodplain meadows are summarised in Table 2. The presence of such features is not always definitive: for simplicity, positive indicators such as ‘possibly’, ‘probably’ etc. have been summed. Meadows entered directly from fords across rivers have been included with droves.

**Table 2:** Presence of diagnostic features (*n* = 373)

		Funnel	Drove	Doles	Common	Drainage
Negative		238	223	271	126	199
Unknown				1	39	
Positive	yes	105	133	77	185	158
	possibly	22	14	9	22	16
	probably		1		1	
	remnant	4		15		
	partial	4				
	ford		2			
Sum of positives		135	150	101	208	174
%positives		36.2%	40.2%	27.1%	55.8%	46.6%

Fieldnames associated with floodplain meadows in the aggregated data are summarised in Table 3. ‘Ing’, ‘mead’ and ‘ham’ elements predominate. Between them, ‘lot’ and ‘dole’ names are reasonably frequent, as is ‘common’.

**Table 3:** Summary of fieldname elements (*n* = 373)

Fieldname element	Includes	Count
*ing*	ing, ings	90
*mead*	mead, meadow	78
*ham*		71
*lot*	lot, allotment, allotments	18
*d?l*	dole, dale	7
*common*		11
*lea*	lease, leaze	5
*hay*	hay, hays	4
*lammas*		3
*ley*		1

This is, as yet, a limited dataset: though it is already greater in number than the 365 existing meadows across England and Wales mapped on the basis of surviving habitat, as shown in January 2025 on the FMP online map^7^. The map of historic floodplain meadows has significant scope to increase, adding further catchments that would increase the baseline of evidence on which we can draw. However, even at this stage, several points of discussion can be tentatively offered.

### Origins

The broad evidence for floodplain meadows discussed above suggests that they may date back to the early medieval period in the form we have identified here, with earlier antecedents for using floodplains for meadows stretching back into prehistory. However, the sources of spatial evidence that we use for mapping are predominantly nineteenth and twentieth century, with some seventeenth and eighteenth-century maps where available. Whilst we can be reasonably confident that spatial evidence from these later centuries enables us to record the form of medieval floodplain meadows embedded in the landscape (and sometimes still in use in the nineteenth century), direct evidence from the medieval period is limited. However, the place-based studies we have carried out so far illustrate the relevance of three sets of medieval sources: the Domesday survey; other documentary sources; and archaeological evidence.

#### Domesday

The Domesday Book details the results of a survey carried out between 1066 and 1086 of land and people in England south of a line from the Tees to the southern edge of the Lake District. The survey captures information about the character, inhabitation, organisation, and use of the early medieval landscape at the time of the Norman Conquest. The Domesday Book catalogues meadows expressly and systematically as one of three key categories alongside ploughland and woodland. In the Domesday Book, ‘meadow often referred to land susceptible to flooding as it was next to water’^8^, hence references to meadows in Domesday can be equated with floodplain meadows. The information compiled in the Domesday Book is thorough and systematic, but Darby (Darby 1986, 13) makes an important caveat:

‘When this great wealth of data is examined more closely, perplexities and difficulties arise. Domesday Book is far from being a straightforward document. It bristles with difficulties.’

Notably, the relationship between manors (ownership) and vills (places that can be mapped, equating to parishes) is complicated: some manors with meadow encompass multiple vills; and some vills are split between multiple manors recorded as having meadow. Consequently, Domesday does not enable robust identification of specific vills with meadow except where one manor equates to one vill. Even at its best, Domesday can only indicate the quantity of meadow in a manor that may, judiciously, be associated with a parish: it does not indicate the physical location of meadows within parishes.

Nonetheless, the relationship between parishes and meadows in Domesday may provide a broad sense of the distribution of floodplain meadow in a catchment. On the Culm, for example, the Domesday record for Columbjohn^9^ states that the settlement had seven acres of meadow compared to three acres of ploughlands; Rewe^10^ also had seven acres of meadow for five acres of ploughlands; and [Monk] Culm^11^ in Silverton had eight acres of meadow for five of ploughlands. The parish boundaries of Rewe, Broad Clyst (including Columbjohn) and Silverton (including [Monk] Culm) all meet in the floodplain, which might suggest floodplain meadows identifiable from later sources in these settlements date back to 1086: perhaps even that the floodplain was apportioned between parishes when their boundaries were established to provide them all with access to floodplain meadows. Equally, the quantity of meadow recorded in Domesday in manors on the Dorset Stour varies along its length, reflecting to some degree the width of the floodplain where it is constrained geologically. Here, Domesday suggests that parishes in some catchments were already maximising their use of the available floodplain for meadows by the end of the early medieval period.

However, it does not follow that Domesday marked the maximum extent of floodplain meadows across the whole country. There are catchments where the area of floodplain far exceeds the identifiable extent of floodplain meadows: the availability of floodplain exceeded the need for hay in 1086, which is a reminder too that the demand for hay and the extent of floodplain meadows is unlikely to have been constant over the many centuries of the medieval period. It seems likely that the extent of floodplain meadow grew as the population grew; but at times, the need for arable may have outstripped the need for hay, causing some land in the floodplain suitable for meadows to be converted to open arable fields which, although apparently rare in the floodplain, are sometimes visible in lidar data (Firth and Firth 2022, fig. 20). As the population fell in the fourteenth century due to the Black Death, it seems likely that the extent of floodplain meadows reduced, perhaps reverting to marshy rough grazing. The relation between population and extent of floodplain meadows may correlate without the causation being clear: population size may have driven demand for hay; but population size may have also enabled the supply of hay makers and herders necessary for floodplain meadows to be created and sustained.

#### Other Documentary Sources

Other documentary sources from the medieval and post-medieval periods have potential to provide insight into changing supply and demand relating to floodplain meadows. As well as being time consuming to identify and examine, however, such documents are usually imprecise about the location and boundaries of floodplain meadows. Nonetheless, the approach described here could offer a means of resolving the location of floodplain meadows to which old texts refer. This possibility prompted us to examine stretches of the Swale, Ure and Ouse, and in Yorkshire, where Hammond (2017; 2021) had previously compiled early (predominantly thirteenth to eighteenth century) references to floodplain meadows that could be linked to parishes, but not to specific floodplain meadows.

The scope to correlate early documentary records with specific floodplain meadows on the Swale, Ure and Ouse was hampered by the catchment having been a focus for enclosure at a relatively early point, which erased the characteristic forms of floodplain meadows before they had time to appear on nineteenth and twentieth century maps. Despite these challenges, mapped landscape evidence could still be used to link documentary references to floodplain meadows compiled by Hammond to their likely former locations. Central to this effort was the term ‘Ing’, a regional term used in Yorkshire for floodplain meadows. A total of 106 floodplain meadows were mapped: over 80 could be related to Hammond’s documented floodplain meadows in some way.

The study showed that research on early documents can be combined with the methodology set out here to locate historic floodplain meadows with a clear degree of confidence. The documentary sources provide additional context and dating, enabling Ings whose location and extent is derived from nineteenth century maps to be extended many centuries earlier, even to the thirteenth century in some cases. In many instances it appears that the floodplain had been enclosed and improved, sometimes behind floodbanks, leaving Ings only as names of rectangular fields that do not represent the earlier extent of floodplain meadows. Yet the presence of at least some floodplain meadows on the Swale, Ure and Ouse with all the characteristic landscape features seen in other catchments suggest that at some point in the past, floodplain meadows in these distinctive forms were more widespread in this catchment.

#### Archaeological Evidence

The approach taken here is predominantly desk-based, so it does not involve physically intrusive investigations that might elucidate the history of floodplain meadows from excavated features, artefacts, palaeoenvironmental evidence, or material suitable for scientific dating. However, desk-based study can still draw on the results of previous field investigations, identified features (including designated heritage assets), topographic features indicated by lidar, and the physical form of the historic environment. Non-intrusive archaeological evidence for the general relationship between floodplain meadows and other medieval features is presented by the physical connection provided by droveways and green lanes between floodplain meadows and settlements with medieval origins. That is to say, floodplain meadows appear frequently to form a core, connected component of the medieval landscapes that still underpin many rural areas.

More specifically, floodplain meadows sometimes lie adjacent to known medieval sites and features, reflecting their respective boundaries and appearing, therefore, to be contemporary with each other. There are multiple instances where legally protected scheduled monuments of medieval date are associated with floodplain meadows, as at Inglesham on the River Cole (Firth and Firth 2022, fig. 45). Instances where medieval settlements, open arable fields and floodplain meadows lie together are especially valuable as they underline the vital role of floodplain meadows to the medieval rural economy alongside the more often recognised and acknowledged ridge and furrow.

Having demonstrated that their distinctive features can be mapped, the approach outlined here will hopefully bring greater attention to the role of floodplain meadows in the medieval landscape, and to their survival and conservation.

### Distribution

Using this method, we have identified floodplain meadows across England and into Wales: in the north west in the Eden catchment; in the north east in the Ouse catchment; in central England in the Thames catchment; in the south west in the Dorset Stour and the Culm; in the Severn and Avon; and in the upper reaches of the Wye, in Wales. The same characteristic features are evident in all these catchments, suggesting that the agricultural practices and governance that gave rise to them were widespread. The ready identification of floodplain meadows on the Eden and the Culm demonstrates that the form is not restricted to the central region or province especially associated with arable open fields (Hall 2014, 2–3; Rippon 2014, 2–7). We are confident that equivalent studies in eastern England would identify floodplain meadows with the same features; studies further afield may identify similar elsewhere^12^ – reflecting the pervasive importance of hay and common meadows to the functioning of rural societies and their economies. The geographical spread and extent of floodplain meadows identifiable using a landscape approach is in any case already greater than that of floodplain meadows based on surviving habitat.

We have noted already – and will discuss further below – that there are other forms of meadows in floodplains: our identification of floodplain meadows does not support the suggestion that all floodplain was managed as common meadow. The identification of historic floodplain meadows is hampered by subsequent land use. Nonetheless, in some catchments, the floodplain meadows we have identified occupy much of the floodplain, especially where the floodplain itself is constrained by the overall topography. In other catchments, especially where the floodplain is very extensive, floodplain meadows seem to occupy only a part of it, the rest of the floodplain perhaps remaining in use as marsh and rough grazing because it exceeded either the demand for hay or the capacity of the local inhabitants to manage it as meadow.

Where meadows lie entirely outside the floodplain, they fall outside the scope of the studies discussed here and have not been mapped or recorded. However, some floodplain meadows appear to lie partly within the floodplain and partly on the slopes above. Even allowing that the extent of modern floodplains may be different to the floodplain when the meadows were established and maintained, some floodplain meadows include areas at too high an elevation to have flooded. As the seasonal addition of nutrients from flooding is regarded as fundamental to floodplain meadows, the presence of non-flooding areas is puzzling. It is possible that in some cases, other hydrological mechanisms such as minor tributaries, groundwater seepages, or springs since intercepted by field drains provided inputs to elevated areas; or perhaps acceptable outcomes were achieved through the dispersal of flood-delivered nutrient by stock or spreading farmyard manure. We have felt it important to include them even if their explanation is currently unsatisfactory.

The suggestion above that even minor tributaries and springs could be adequate drivers for floodplain meadows is important: floodplain meadows are not limited to the extensive floodplains of the middle and lower reaches of catchments. Our approach has probably under-represented floodplain meadows in upper reaches because our work has concentrated on the main channels of watercourses. Taking in the topmost headwaters represented by tiny rivulets would significantly increase the area of investigation, acknowledging also that watercourses in headwaters are either too small to be represented in historical sources or have already been straightened or replaced by field drains by the time that detailed large scale mapping becomes available. However, we have identified examples of floodplain meadows on small streams relatively high in catchments for example, the floodplain meadow at Ferny Piece Copse on the Bolham River in the Culm catchment^13^. Although these floodplain meadows may be narrow and sinuous, they still have features consistent with the origins of much broader floodplain meadows further downstream and were no doubt important to upland agriculture.

**Fig. 5:**
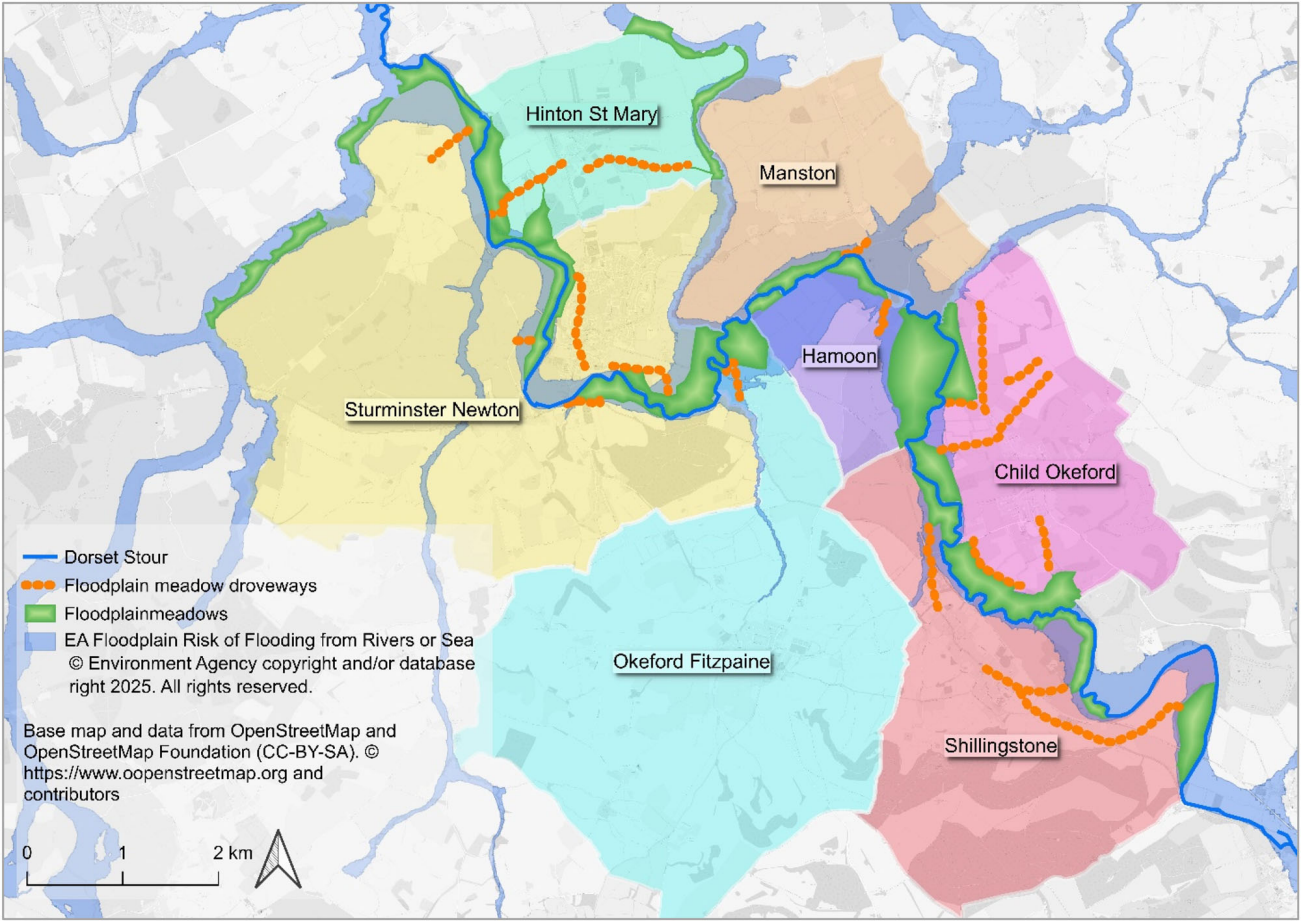
Floodplain meadows (green tint) along the Dorset Stour, with droveways (orange dots) and parishes

Our initial identification of floodplain meadows and the method set out here are based on the articulation between floodplain meadows and the agricultural system that gave rise to them: notably the funnel-shaped boundaries where they attach to droves and green lanes that connect them to settlements and other parts of the landscape (Fig. 5). The interdependence between floodplain meadows and their economic and social setting was expressed physically in their boundaries, in the droves providing their access, and in the sources that map them. Insofar as other boundaries respect them, it appears that meadows and droves are some of the earliest features of our current landscape. As noted above, it seems that the floodplain of the Culm near Killerton, for example, was apportioned between parishes (by agreement or dispute) to ensure each had access to floodplain meadows. The presence of routes deeply embedded in the landscape suggest that flows of animals, of workers, and of hay were as important to the functioning of floodplain meadows as flows of water and nutrients. Links to drier land and to settlement are intrinsic, therefore, to the distribution and common governance of floodplain meadows, which still seem to be associated with historic paths and rights of way as well as droveways. The distribution that we see across multiple catchments suggests that floodplain meadows are not just a consequence of the organisation of agricultural society that we see in the landscape, but perhaps one of its main drivers.

### Decline

Despite their importance for producing hay, floodplain meadows were subject to the pressure of successive waves of enclosure and agricultural intensification. This manifests itself in different ways. In some cases, common meadows were enclosed in the sense that they ceased to be common but retained their form to the present. In other cases, there is little trace of their earlier form, earlier boundaries having been completely removed and replaced by patterns of rectilinear fields: the former presence of a floodplain meadow indicated only by field names and the modern risk of flooding. Between these lie a range of circumstances, including instances where the strip system of doles has transformed to field boundaries progressively encroaching on a diminishing floodplain meadow. Field boundaries along Southfield Lane off Hayward Lane^14^, Childe Oakford on the Dorset Stour, offer an example. Encroachment hinders the identification and mapping of floodplain meadows from later sources and reduces the confidence that can be placed on their interpretation. It suggests chronological differences too: the difficulty of identifying floodplain meadows in parts of the Ouse catchment reflecting the early enclosure of that landscape, for example; and the transformation of doles into bounded fields perhaps indicating earlier piecemeal enclosure whilst the survival of private but entire floodplain meadows represents planned parliamentary enclosure in later periods.

Enclosure and the transformation of common into private management is likely to have gone hand in hand with historic ‘improvement’, some of which is visible in mapped sources such as the canalisation of rivers and tributaries resulting in straight channels and drains. Instances of former floodplain meadows being replaced by bedworks are apparent at multiple places in the Thames tributaries, such as Town Mead, Highworth on the River Cole (Firth and Firth 2022, 71)^15^; the floodplain meadow at Little Hayward on the river Trent near Shugborough was partly encroached upon by bedworks, as indicated in Fig. 4. Despite enthusiasm for bedworks, however, this progression was not universal. There are many instances – as Fig. 4 shows – where floodplain meadows persisted in their early form well into periods when bedwork systems were being built nearby. On the Dorset Stour, River Culm and others, doles on tithe maps indicate that floodplain meadows were still in use c. 1840 in catchments with extensive bedworks. In such instances, dole systems were maintained on floodplain meadows that had been taken into private ownership – a necessary precursor to the influx of capital required to construct bedwork systems – without then turning into bedworks. Floodplain meadows may have persisted alongside bedwork systems because they were sufficiently productive without capital investment, or so central to rural society that they could not be disrupted.

Other forms of intensification are less visible: sub-surface drainage, conversion to arable, re-seeding with ryegrass, introduction of artificial fertilizer. Some major, visible changes were not driven by agricultural intensification, but by other forms of development. On the Culm and Wye, for example, railway construction in the nineteenth century had a direct impact on floodplain meadows; but perhaps more pervasive consequences by severing access, altering the floodplain with their embankments, interfering with drainage and changing patterns of ownership.

The varying history of floodplain meadows and their many changes since their presumed peak in the twelfth to fourteenth century means that they cannot be simply digitised from early maps. Even by the time these maps were prepared in the eighteenth and nineteenth centuries, some floodplain meadows had been taken out of common management into private ownership, had been replaced by bedworks, or had new boundaries imposed through incremental or wholescale reorganisation including for permanent pasture or arable use. Nonetheless, the method described here can see through these later changes to postulate the original extent of floodplain meadows even where their long history came to an end before they could be mapped directly. It is possible to use eighteenth and nineteenth century sources to investigate much older landscapes.

### End?

Aside from those that exist today, as mapped by FMP based on surviving habitat^16^, floodplain meadows were replaced over the centuries. As noted above, floodplain meadows were replaced by bedworks, encroached upon or obliterated by enclosure (physically, and in terms of ownership and governance), or affected by development such as railway construction. In some cases, however, their form survived even though the practices that maintained their habitats largely ceased. Identifying their surviving form using landscape sources does not provide details or chronology for the intensification that extinguished their habitats, such as introduction of field drainage, switching to arable, the application of artificial fertilizers, or re-sowing for intensive pasture. It seems likely that traditional management of floodplain meadows ceased in tandem with bedwork watermeadows going out of use in the agricultural depression of the late nineteenth century (Historic England 2018, 4), coupled with the introduction of mechanisation in farming, industry and transport which reduced the need for winter fodder, such as hay. The loss of habitat likely accelerated through the twentieth century contributing to the decline in species-rich grassland outlined in the introduction. As noted, intensive practices did not always result in physical changes to boundaries and other features, hence the boundaries of floodplain meadows still survive today even though the habitat and the practices that gave rise to them are long gone. The form of floodplain meadows – including their associated droves – survives in many of the catchments we have examined, and are visible on OS maps and especially on satellite imagery. These survivals might offer especially evocative opportunities for restoration, returning centuries-old practices to places whose form still reflects that history. Even where their original form has been compromised partly or entirely by enclosure, the evidence for former floodplain meadows might still offer firmer ground for restoration than environmental suitability alone.

## Implications for Restoration

This work is relevant to the restoration of floodplain meadows on two levels: strategic, and place based. At a strategic level, these investigations can inform estimates of the original extent—and consequently the loss—of floodplain meadow habitats at a national, regional, county or catchment scale. They can help in quantifying the significance of remaining habitat, identifying potential opportunities for landscape scale restoration and recovery, and informing local plans for nature restoration. Also at a strategic level, this work can inform policy, drawing attention to an overlooked form of sustainable, regenerative agricultural practice and supporting the case for specific indices and mechanisms that will incentivise its restoration and maintenance. There are general opportunities too to raise awareness among stakeholders in agriculture, land management, catchment management and nature conservation, about the scale and former importance of floodplain meadows. For example, this work is a reminder that floodplains have been sustained by grassland agriculture rather than woodland for at least a thousand years and probably longer. Engagement with practitioners might also emphasise floodplain meadows as the outcome of re-establishing (agri)cultural practices rather than as a thing that can be reconstructed through a short phase of capital works. The importance we have flagged here of connectivity, access and governance is worth emphasis with practitioners too: the history of floodplain meadows suggests that restoration measures that impede mowing or render the removal of baled hay impractical will limit success; but there are historical grounds for increasing public accessibility and the role that meadows played in networks of footpaths and rights of way. Public engagement itself offers a further strategic value to this approach: the long history of floodplain meadows, their common governance, the human character of the practices that sustained them, and their wide distribution provide an engaging platform to raise public awareness of the importance of floodplain restoration. To the benefits of floodplain meadows in terms of flood risk management, carbon sequestration, water-quality and nature recovery might be added the contribution of their history to wellbeing and community resilience^17^.

A further way in which this work has strategic value is in raising the importance of floodplain meadows in understanding the history of landscape and agriculture. The history of floodplain meadows appears to have received little scholarly attention, seemingly overshadowed by the history of arable fields and bedwork watermeadows with their humps and bumps: floodplain meadows are mentioned only in passing, presumed to lie on the margin of agricultural life rather than at the centre. Although the work set out here has concentrated quite narrowly on sources indicating spatial extents, it raises a wider range of questions about their origin, function, governance, distribution, chronology and so on that other sources will inform or challenge.

The relevance of this work to place-based activities stems from the detail that this approach provides on the location, extent, context and connectivity of specific floodplain meadows: including floodplain meadows already mapped, identified and recorded in the catchments so far considered, but also all those to which this approach might be applied elsewhere. The identification of former floodplain meadows might suggest suitable sites for restoration, providing a level of opportunity mapping that can be combined with information about hydrology, soils and ownership. Where features of the floodplain meadow such as boundaries, back drains and funnel-shapes have survived, they are likely to add a sense of authenticity and continuity in the landscape, as well as bringing ancient features back into use as we might with derelict historic buildings. As with such buildings, attention to surviving features might reveal some hidden gems: species or patches of habitat that have hung on despite intensification, with potential to recolonise during floodplain meadow restoration. Other forms of evidence might also be revealed, such as examples of the stones that once marked out systems of doles: the survival of stones and posts is sometimes noted on large scale OS maps from the late nineteenth century; and examples have been found on the ground in some cases^18^.

Place-based research informed by this approach could also start to address questions about the chronology, development and decline of specific floodplain meadows. The sources combined here could be used to identify ditches or infilled palaeochannels likely to contain suitable deposits for core sampling, palaeoenvironmental analysis, and scientific dating. These can provide firm evidence of former hydrology and vegetation and the dates at which they changed, enabling interpretation of the sequence of natural and especially human processes that gave rise to floodplain meadows. Relatively precise historical spatial data can also invigorate research into documentary sources, flagging new lines of enquiry or providing context that makes more sense of previously known references.

The evidence about specific places that this research provides can also inform engagement and discussion with practitioners, stakeholders and the wider public. Robust, transparent data about the presence and history of former floodplain meadows can help to ground conversations on practical details such as boundaries, access and previous management, but also assist in inspiring and envisioning a future that is quite different to the present but validated by the past. The range of historical documents, maps and other sources collated in the studies set out here have obvious value as evidence in supporting discussions about restoration; but they are also great media for engaging with the public through onsite signage, offsite displays, or online guides. There may be opportunities to reintroduce historical information to the landscape by using forgotten field names referring to floodplain meadows, for example.

It is too early to say how this research into floodplain meadows will have consequences for the conservation of floodplain meadow habitats, or for floodplain meadows as tangible components of the historic environment. The approach has, however, already informed planning applications for nature-based solutions at Killerton, for example (Firth 2021); and plans for Stour Valley as strategic greenspace adjacent to the conurbation of Bournemouth, Poole and Christchurch (Firth and Firth 2023a). FMP regularly uses historic evidence in engaging with farmers and land managers about the potential for restoration, especially in catchments such as the Dorset Stour where there is no surviving floodplain meadow habitat but the historical record demonstrates that floodplain meadows were once extensive. The results of this approach are also a major strand of the evidence underpinning the floodplain meadow restoration strategy for the Windrush (Cotswolds National Landscape et al. 2024). There is good reason to hope that this research will lead to a reappraisal of the importance of floodplain meadow habitats and their historic landscape forms in the wave of initiatives and funding being directed in the UK at nature, landscape recovery, and sustainable farming.

A final point to note here is the primacy of evidence, and professional care over its interpretation. In all instances where historic floodplain meadows are identified, a degree of inference is required. Although engaging, the sources do not speak for themselves. In some cases, as indicated above, the sources are thin and the inferential leaps quite large in the identification of a floodplain meadow, or in the identification of its boundaries and features. Overall, we have been cautious in our approach and have expressly documented levels of confidence. Given likely effacement by bedworks, the presence of private meadows in the floodplain, encroachment from enclosure and historic ‘improvement’, and our reluctance to step too far from the evidence, then the floodplain meadows that we have mapped probably underrepresent their former extent. This is preferable to making unsupported assertions about the character of our countryside and its past, reshaping its future on nebulous grounds; but to embark on nature-based solutions and nature recovery in floodplains without expressly engaging with the cultural heritage of floodplain meadows would be more unfortunate still.

## Conclusions

The methodology set out here has been successful in identifying and mapping the historic extent of floodplain meadows. The results enable the relationship between floodplain meadows and parishes and settlements to be explored.

The approach shows that floodplain meadows that were managed in common persist in the landscape, even where they are previously unrecorded. In some cases, their form and topographic features survive, where the habitat has been extinguished by historic ‘improvement’ or intensive agriculture. Even where they do not survive physically, their presence can be traced in other sources, such as historic maps, documents and archive data. However, old maps do not speak for themselves. By the time the first large scale, precise maps were made – such as tithe maps and OS maps – floodplain meadows had already disappeared in some places due to enclosure or other encroachment. In these cases, identification of former floodplain meadows requires interpretation based on multiple sources.

The sources used in this methodology represent only a few data points referring to specific points in time: caution is required about the weight placed on just a few maps and geographical accounts. Notwithstanding, the sources are sufficient for the earlier extent of floodplain meadows to be mapped in many catchments. A robust and transparent method has been developed for evidencing the former presence and extent of floodplain meadows locally and regionally. This offers a way of quantifying loss and rarity; but it is also a way of flagging potential sites for restoration, especially where physical features such as boundaries still survive. Moreover, the method provides a means of directly integrating restorable meadows into catchment management alongside other opportunities for nature-based solutions.

This work emphasises that floodplain meadows are not just physical things: they embody cultural practices and comprise both tangible and intangible heritage. Their forms manifest practices carried out over many generations, presenting Traditional Ecological Knowledge that is embodied in – and interpretable from – the landscape itself. The character of floodplain meadows as practices is underlined by their demonstrable relationship with the parishes and settlements that gave rise to them, their common governance, and the droves that were key to their accessibility and gave them their characteristic form. Although floodplain meadows have a distinct chronological and geographic context, this approach is surely relevant across the globe to the restoration of anthropogenic habitats where traces interpretable from the historic landscape are more extensive than the survival of the habitat itself.

The potential of this methodology for supporting public engagement has also been demonstrated, by adding to the historical dimension of floodplain meadows and especially their local context; whilst also flagging directions that could be taken by community groups in investigating their own floodplain meadows.

The methodology supports the view that floodplain meadows were both widespread across England and Wales and central to the organisation of the rural economy for a thousand years: their geographic span – and the potential of the approach outlined here – may have matched the extent of medieval open field agriculture in Europe. Common governance, co-creation and public access were fundamental to floodplain meadows in the past, and are perhaps fundamental also to their restoration and maintenance in future. With their forms still embedded in the landscape, floodplain meadows offer valuable lessons as we seek to re-establish resilient habitats, places and communities.

## Data Availability

The digital map generated by this study is available online at https://floodplainmeadows.org.uk/discover/learn/history/historic-sites-map. The dataset is available on request from the Floodplain Meadows Partnership.

## Footnotes

1 European Environment Agency, 2019. Habitat factsheets E2.14 and 3.4a. https://eunis.eea.europa.eu/habitats/1635; https://eunis.eea.europa.eu/habitats/8124.

2 ‘Heritage Asset: A building, monument, site, place, area or landscape identified as having a degree of significance meriting consideration in planning decisions, because of its heritage interest.’ (Ministry of Housing, Communities and Local Government 2023, 70).

3 Halberton, c1605: Devon Heritage Centre, reference number 6065Z. https://devon-cat.swheritage.org.uk/records/6065Z.

4 https://eunis.eea.europa.eu/habitats/2456.

5 https://floodplainmeadows.org.uk/discover/learn/history/historic-sites-map.

6 A further 18 have been identified at Shugborough on the Rivers Sow and Trent, and 82 on the upper reaches of the Wye, which will be added to the online map in due course.

7 https://floodplainmeadows.org.uk/discover/floodplain-sites-map.

8 National Archives https://www.nationalarchives.gov.uk/domesday/world-of-domesday/landscape.htm.

9 https://opendomesday.org/place/SX9599/columbjohn/.

10 https://opendomesday.org/place/SX9499/rewe/.

11 https://opendomesday.org/place/SS9502/monk-culm/.

12 An initial study (Firth and Firth 2025) indicates the presence of floodplain meadows with the same diagnostic characteristics on the Rivers Dee, Usk, and Teifi in Wales, in addition to the upper reaches of the Wye.

13 https://floodplainmeadows.org.uk/node/13585.

14 https://floodplainmeadows.org.uk/node/13636.

15 https://floodplainmeadows.org.uk/node/13666.

16 https://floodplainmeadows.org.uk/discover/floodplain-sites-map.

17 https://historicengland.org.uk/research/results/reports/18-2024.

18 https://floodplainmeadows.org.uk/sites/default/files/files/Floodplain%20Meadows%20-%20Beauty%20and%20Utility%20A%20Technical%20Handbook.pdf p. 11, 14, 16-18.
